# Supplementary material for: Genomic mutation landscape of skin cancers from DNA repair-deficient xeroderma pigmentosum patients
Source: Nat Commun. 2023 May 4;14:2561. doi: 10.1038/s41467-023-38311-0 (PMC10160032; doi:10.1038/s41467-023-38311-0)
Supplement: Supplementary file 1 — Supplementary Information [file 41467_2023_38311_MOESM1_ESM.pdf]

GENOMIC MUTATION LANDSCAPE OF SKIN CANCERS FROM DNA REPAIR-DEFICIENT XERODERMA PIGMENTOSUM PATIENTS

Yurchenko et al.

Supplementary Table 1. Xeroderma Pigmentosum tumors used in the analysis.

Patient	Group	Cancer type	Material	Source	Germline mutation	Sample origin	Tumor coverage, X	Normal coverage, X	Tumor purity %
CN001	XP-A	BCC	FFPE	This study	XPA:NM_000380:exon6:c.C682T:p.R228X, NM_000380:exon4:c.390-1G>C (splice-site)	Japan	19	17	28
CN001	XP-A	SCC	FFPE	This study	XPA:NM_000380:exon6:c.C682T:p.R228X, NM_000380:exon4:c.390-1G>C (splice-site)	Japan	14	17	23
CN001	XP-A	BCC	FFPE	This study	XPA:NM_000380:exon6:c.C682T:p.R228X, NM_000380:exon4:c.390-1G>C (splice-site)	Japan	17	19	32
PD37450a	XP-C	SCC	Fresh	Momen et al. 2019	XPC:NM_004628:exon4a:c.445_446delGA:p.G149fs, exon13:c.2336_delT:p.L779fs	UK	35	31	35
SRR2194572	XP-C	SCC	Fresh	Zheng et al. 2014	XPC:NM_004628:exon13:c.2251-1G>C (splice-site)	UK	22	10	89
SRR2194573	XP-C	SCC	Fresh	Zheng et al. 2014	XPC:NM_004628:exon8:c.940delC:p.R314fs	UK	13	8	84
SRR2194574	XP-C	SCC	Fresh	Zheng et al. 2014	XPC:NM_004628:exon8:c.940delC:p.R314fs	UK	38	14	72
CM002	XP-C	MEL	Fresh	This study	NM_004628:exon7:c.780-2A>T (splice-site), NM_004628:exon5:c.621+1G>A (splice-site)	Brazil	60	23	30
HF001	XP-C	SCC	FFPE	This study	XPC:NM_004628:exon6:c.C658T:p.R220X (stopgain)	UK	14	36	34
HF010	XP-C	BCC	FFPE	This study	XPC:NM_004628:exon14:c.2429_2441del:p.G810fs, NM_004628:exon2:c.299+2T>- (splice-site)	UK	25	40	30
SA006	XP-C	SCC	FFPE	This study	XPC:NM_004628:exon9:c.1643_1644del:p.V548fs	France	30	26	46
RC12-032T	XP-D	SCC	Fresh	Cho et al. 2018	ERCC2:NM_000400:exon22:c.C2047T:p.R683W	UK	54	42	29
HF006	XP-D	BCC	FFPE	This study	ERCC2:NM_000400:exon22:c.C2047T:p.R683W	UK	41	42	28
HF008	XP-D	SCC	FFPE	This study	ERCC2:NM_000400:exon22:c.C2047T:p.R683W, NM_000400:exon10:c.816-2A>G (splice-site)	UK	28	34	31
HF002	XP-E	SCC	FFPE	This study	DDB2:NM_000107:exon4:c.458delT:p.I153fs, NM_000107:exon6:c.C820T:p.Q274X (stopgain)	UK	33	36	57
SA001	XP-E	MEL	FFPE	This study	DDB2:NM_001300734:exon4:c.G550C:p.D184H	France	40	46	49
SA001	XP-E	BCC	FFPE	This study	DDB2:NM_001300734:exon4:c.G550C:p.D184H	France	57	46	20
SA001	XP-E	BCC	FFPE	This study	DDB2:NM_001300734:exon4:c.G550C:p.D184H	France	7	46	36
TP001	XP-E	SCC	Fresh	This study	DDB2:NM_001300734:exon4:c.G460C:p.A154P	Brazil	32	27	27
TP001	XP-E	SCC	Fresh	This study	DDB2:NM_001300734:exon4:c.G460C:p.A154P	Brazil	38	27	45
TP001	XP-E	SCC	Fresh	This study	DDB2:NM_001300734:exon4:c.G460C:p.A154P	Brazil	31	27	30
TP001	XP-E	SCC	Fresh	This study	DDB2:NM_001300734:exon4:c.G460C:p.A154P	Brazil	34	27	35
TP002	XP-E	SCC	Fresh	This study	DDB2:NM_001300734:exon4:c.G460C:p.A154P	Brazil	33	34	20
TP002	XP-E	SCC	Fresh	This study	DDB2:NM_001300734:exon4:c.G460C:p.A154P	Brazil	34	34	34
AS002	XP-V	MEL	FFPE	This study	POLH:NM_001291969:exon8:c.719dupC:p.T240fs	France	52	38	36
AS003	XP-V	BCC	FFPE	This study	POLH:NM_001291969:exon6:c.C535T:p.R179X (stopgain), POLH:NM_001291969:exon8:c.849_852del:p.N283fs	France	58	38	32
AS004	XP-V	MEL	FFPE	This study	na	France	84	35	38
CM003	XP-V	MEL	Fresh	This study	POLH:NM_001291969:exon6:c.C535T:p.R179X	Brazil	42	23	61
CM004	XP-V	BCC	Fresh	This study	NM_001291969:exon4:c.392+1G>A (splice-site),POLH:NM_001291969:exon6:c.C535T:p.R179X	Brazil	59	28	30
CM006	XP-V	BCC	Fresh	This study	NM_001291969:exon4:c.392+1G>A (splice-site)	Brazil	57	25	34
CM006	XP-V	BCC	Fresh	This study	NM_001291969:exon4:c.392+1G>A (splice-site)	Brazil	58	25	54
CM006	XP-V	BCC	Fresh	This study	NM_001291969:exon4:c.392+1G>A (splice-site)	Brazil	59	25	36
CM008	XP-V	BCC	Fresh	This study	NM_001291969:exon4:c.392+1G>A (splice-site)	Brazil	33	24	51
CM008	XP-V	BCC	Fresh	This study	NM_001291969:exon4:c.392+1G>A (splice-site)	Brazil	60	24	21
CM011	XP-V	BCC	Fresh	This study	POLH:NM_001291969:exon8:c.849_852del:p.N283fs	Brazil	60	27	83
CM011	XP-V	BCC	Fresh	This study	POLH:NM_001291969:exon8:c.849_852del:p.N283fs	Brazil	59	27	71
CR006	XP-V	BCC	Fresh	This study	POLH:NM_001291969:exon9:c.1353delC:p.V451fs	France	35	40	54
CR007	XP-V	BCC	FFPE	This study	POLH:NM_001291969:exon6:c.C535T:p.R179X (stopgain), NM_001291969:exon8:c.849_852del:p.N283fs	France	79	39	16

Supplementary Table 2. Summary of RPE-1 cell line experiments.

Cell line	POLH status	Treatment	clone	SBS	DBS	CC>TT	SBS per MB	Mean coverage
RPE_1	POLH_KO	KbrO	D_1	57349	273	0	22.314786	16.628305
RPE_1	POLH_WT	KbrO	D_2	47697	109	1	18.559144	16.927795
RPE_1	POLH_KO	NT	D_2	1029	81	4	0.40038911	17.437936
RPE_1	POLH_WT	NT	D_1	1268	18	0	0.49338521	17.406125
RPE_1	POLH_KO	UVA	D_1	13085	215	25	5.09143969	11.161277
RPE_1	POLH_KO	UVA	D_2	16252	296	21	6.32373541	9.510871
RPE_1	POLH_KO	UVA	D_3	17000	279	18	6.61478599	17.200994
RPE_1	POLH_WT	UVA	D_1	2038	33	1	0.79299611	11.49929
RPE_1	POLH_WT	UVA	D_2	2173	47	4	0.84552529	11.139115
RPE_1	POLH_WT	UVA	D_3	2586	29	0	1.00622568	17.196142
RPE_1	POLH_KO	UVC	D_1	606441	16948	8423	235.969261	11.075572
RPE_1	POLH_KO	UVC	D_2	530082	16329	9195	206.257588	10.695095
RPE_1	POLH_KO	UVC	D_3	773036	22243	12006	300.792218	17.15257
RPE_1	POLH_WT	UVC	D_1	57043	1322	479	22.1957198	10.489356
RPE_1	POLH_WT	UVC	D_2	59439	1702	724	23.1280156	11.252983
RPE_1	POLH_WT	UVC	D_3	65761	1722	674	25.5879377	12.743655

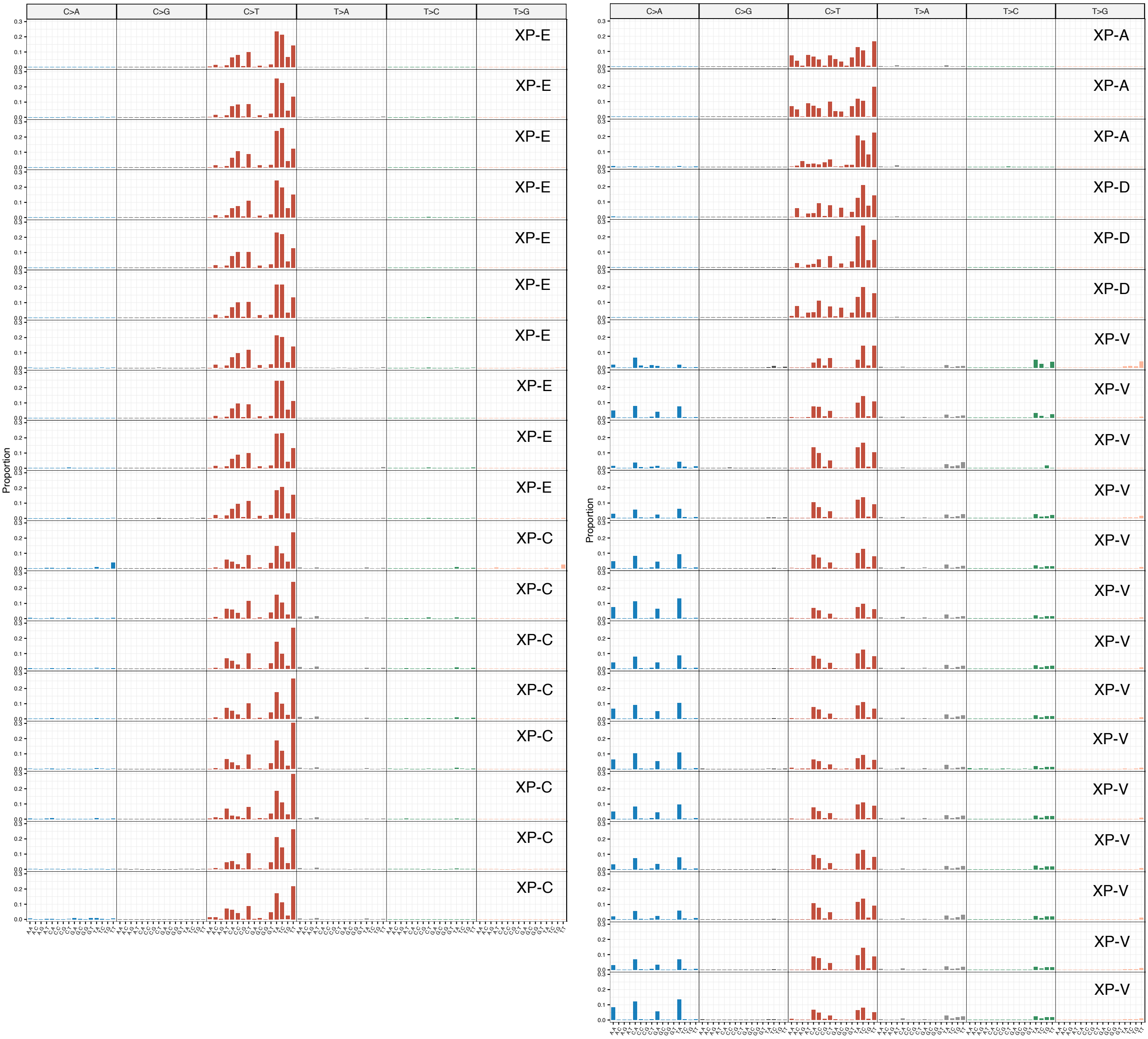

Supplementary Figure 1. Trinucleotide-context mutation profiles of SBS for each tumor from XP patients.

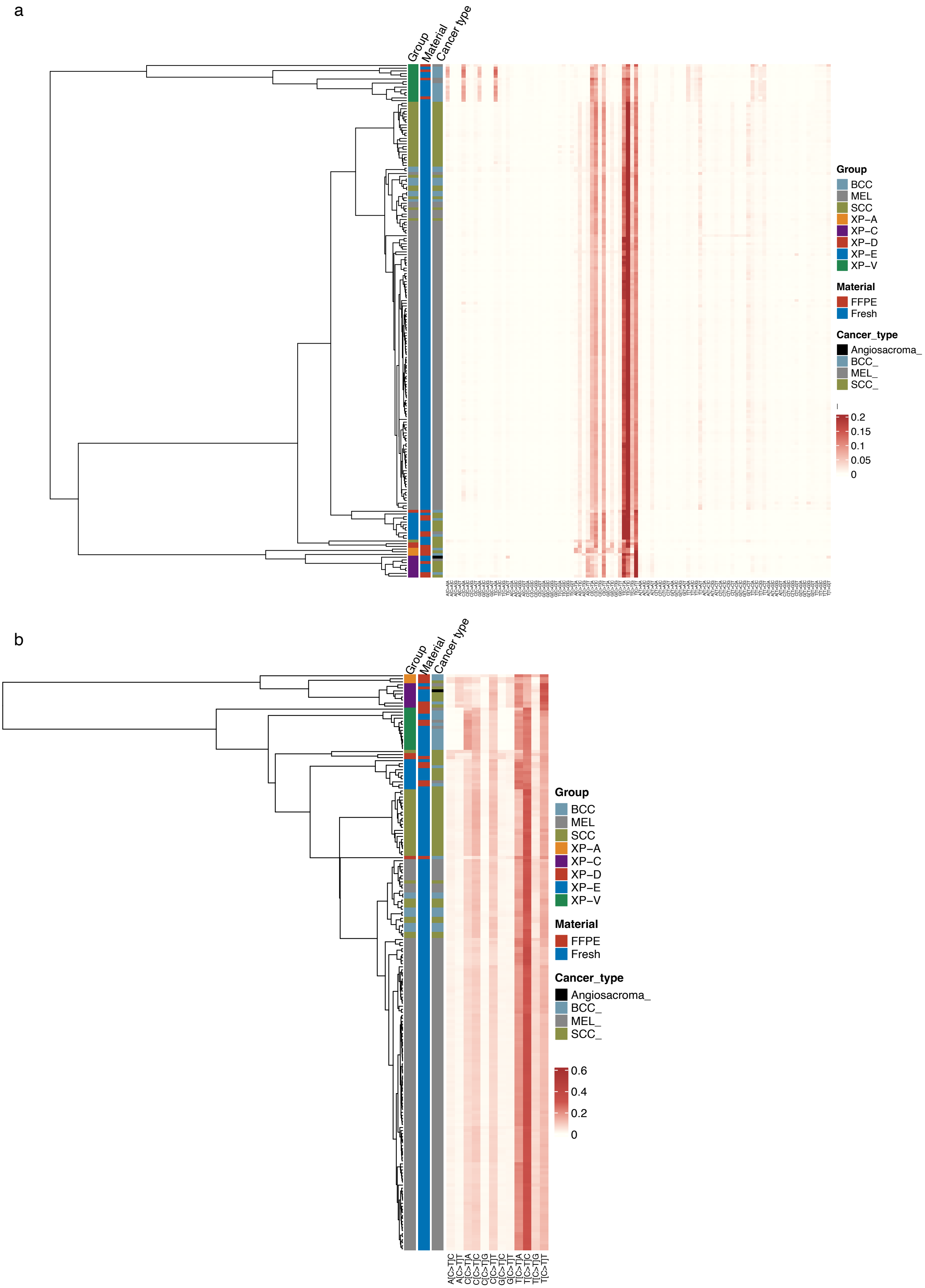

Supplementary Figure 2. Hierarchical clustering of the samples based on the Cosine similarity distance between mutational profiles.
a 96-chanell SBS mutational profiles and all the samples.
b only C>T mutations with adjacent pyrimidines for all the samples.

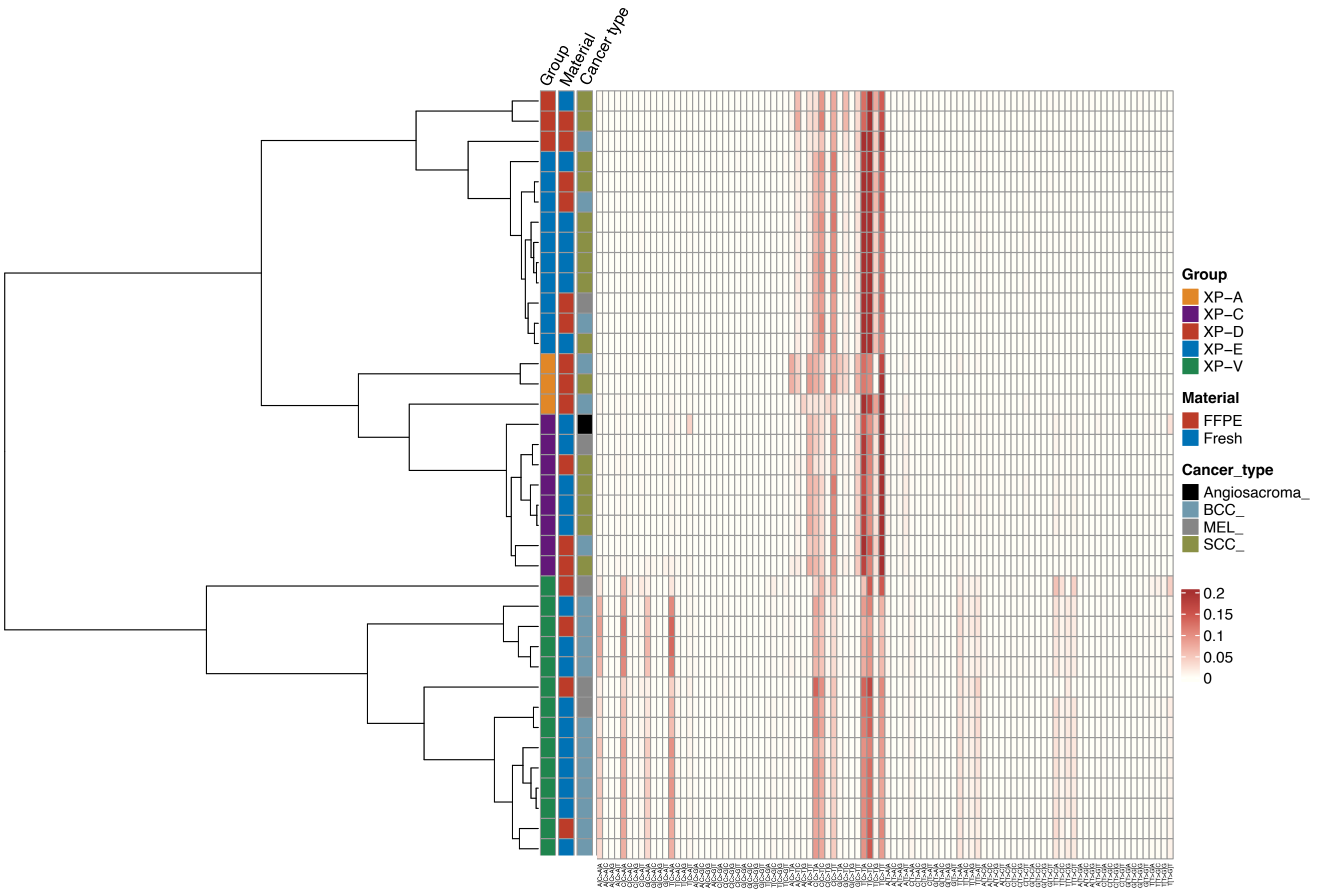

Supplementary Figure 3. Hierarchical clustering of the XP samples based on the Cosine similarity distance between mutational profiles (96-chanell SBS mutational profiles).

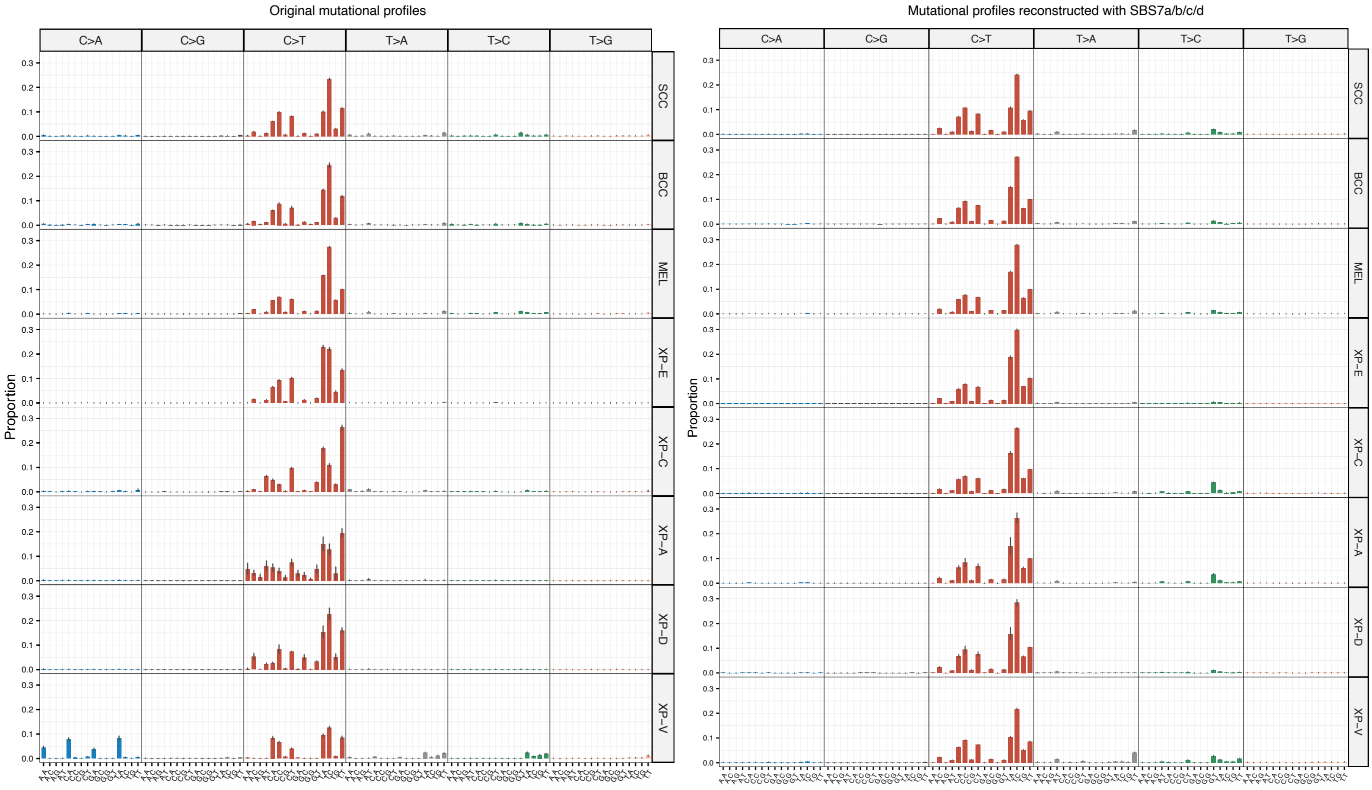

Supplementary Figure 4. Trinucleotide-context mutation profiles of SBS per group for original and reconstructed mutational profiles with COSMIC SBS7a/b/c/d mutational signatures. Data are presented as mean values +/- SEM. Sample size for all the panels (tumors): n = 31 for SCC, n = 8 for BCC, n = 113 for MEL, n = 10 for XP-E, n = 8 for XP-C, n = 3 for XP-A, n = 3 for XP-D and n = 14 for XP-V.

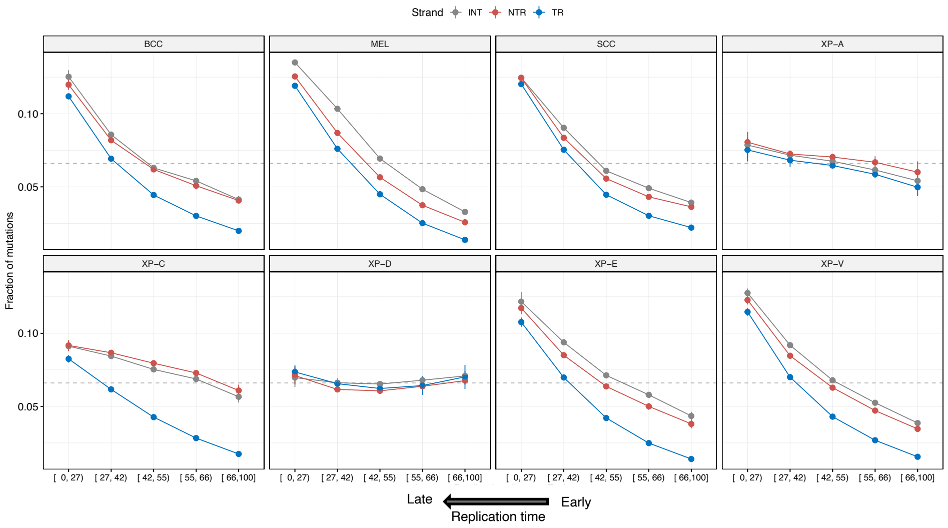

Supplementary Figure 5. Fractions of C>T mutations from pyrimidine dimers in intergenic regions (INT, grey color), on the untranscribed (NTR, red color) and transcribed (TR, blue color) DNA strands of gene regions grouped in 5 equal size bins by replication timing (RT) for XP groups and sporadic skin cancers. Data are presented as mean values +/- SEM. Sample size (tumors): n = 31 for SCC, n = 8 for BCC, n = 113 for MEL, n = 10 for XP-E, n = 8 for XP-C, n = 3 for XP-A, n = 3 for XP-D and n = 14 for XP-V.

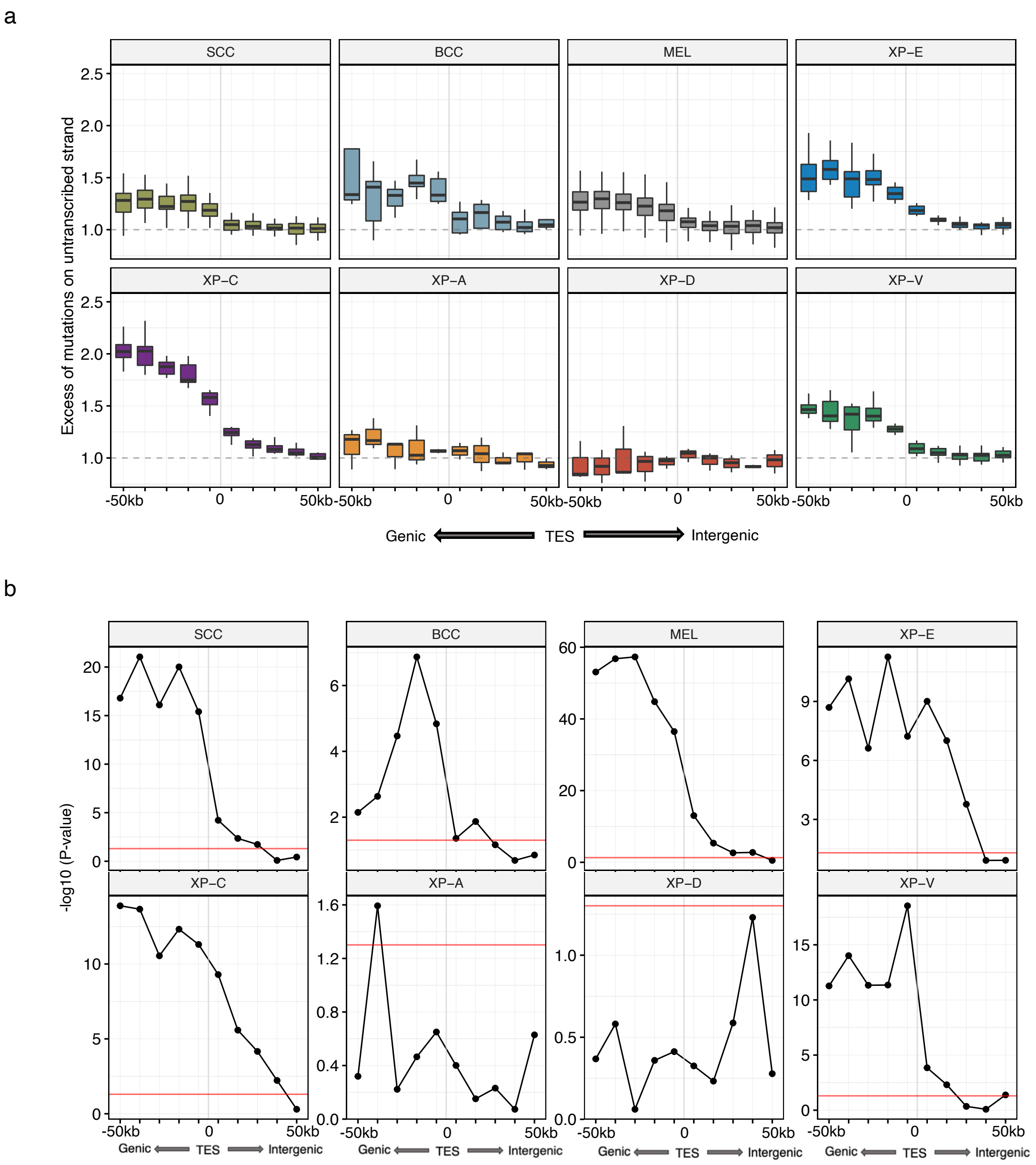

Supplementary Figure 6. Transcriptional bias in the TES-centered 100kb region

a Transcriptional bias (binned by 10kb intervals). Boxes depict the interquartile range (25–75% percentile), lines - the median, whiskers - 1.5× the IQR below the first quartile and above the third quartile. Sample size (tumors): n = 31 for SCC, n = 8 for BCC, n = 113 for MEL, n = 10 for XP-E, n = 8 for XP-C, n = 3 for XP-A, n = 3 for XP-D and n = 14 for XP-V.

b Significance of transcriptional bias (-log10 P-value) in the TES-centered 100kb region (binned by 10kb intervals). Significance was assessed with Welch two sample t-test, two-sided. Red line indicates alpha value 0.05.

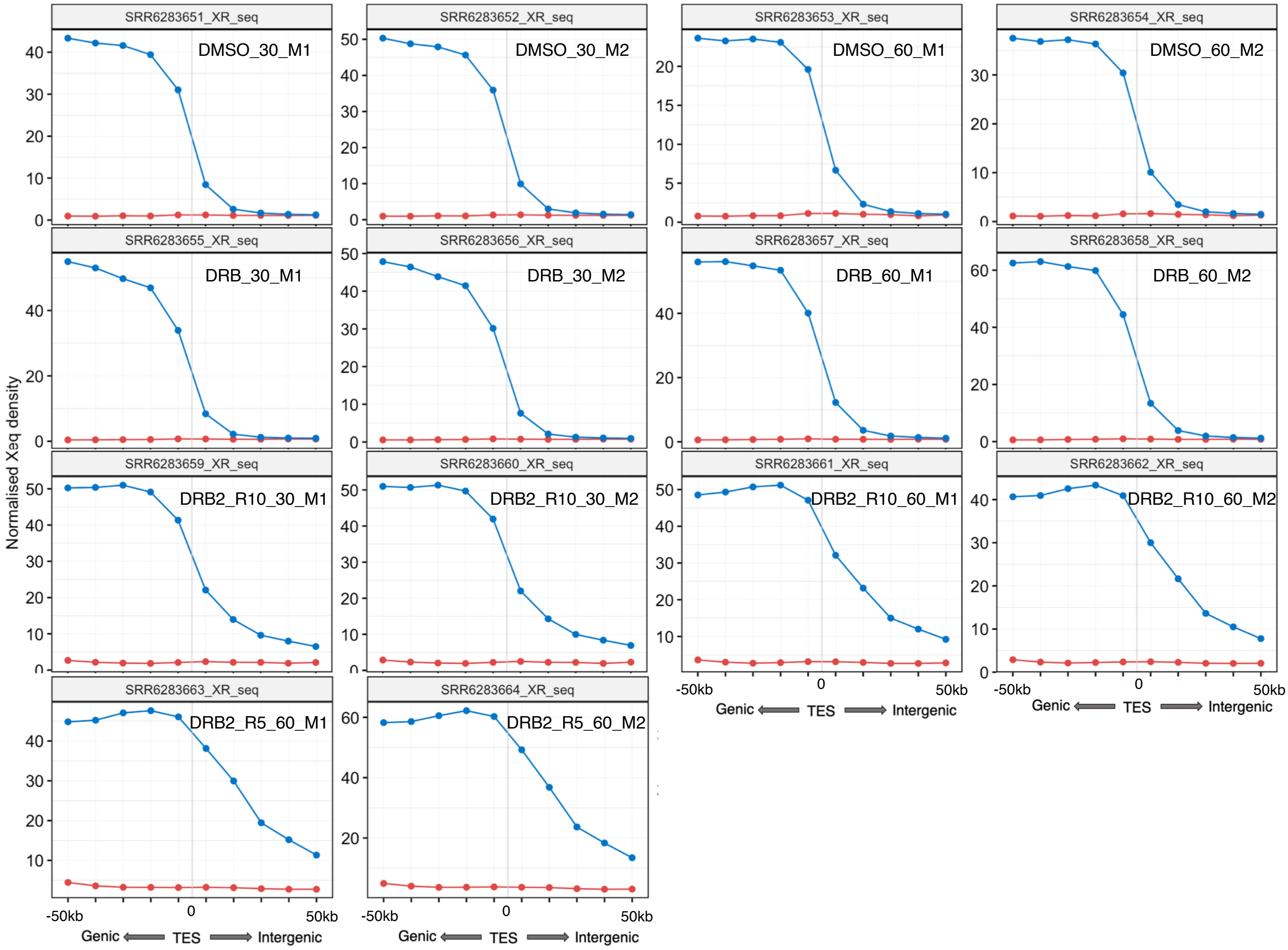

Supplementary Figure 7. DNA context-normalized XR-seq density from XP-C cell line (Chiou et al. 2018 J. Biol. Chem) on untranscribed (NTR) and transcribed (TR) gene strands in the TES-centered 100kb region (binned by 10kb intervals; left panel). DMSO: XR-seq after 30 or 60 minutes after UV irradiation and with DMSO; DRB: XR-seq after 30 or 60 minutes after UV irradiation and DRB (transcription inhibitor 5,6-dichlorobenzimidazole 1-β-d-ribofuranoside) treatment; DRB2: XR-seq after 30 or 60 minutes after UV irradiation and DRB treatment before and after exposure. For each panel n = 1.

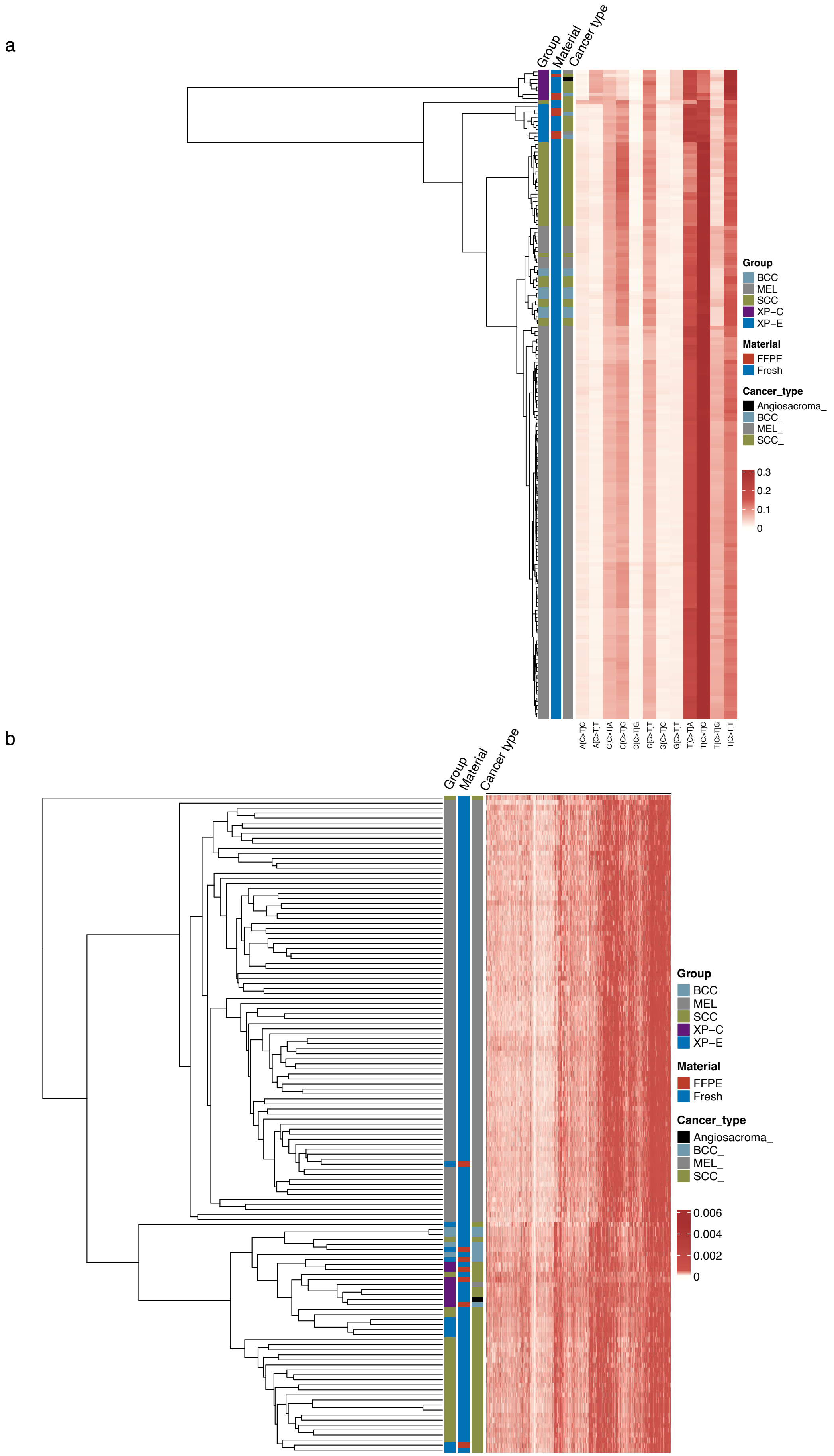

Supplementary Figure 8. Clustering of mutational profiles for XP-C, XP-E and sporadic cancers.
a Unsupervised clustering plot based on the Cosine similarity distance between the SBS trinucleotide-context mutation profiles of the samples (only C>T mutations with an adjacent pyrimidine (YC>YT or CY>TY), the typical UV mutation context).
b Unsupervised clustering plot based on the density of mutations in 2684 1Mb-long windows along the genome (only for samples with more than 50k mutations belong to sporadic, XP-C and XP-E groups; WPGMA clustering method).

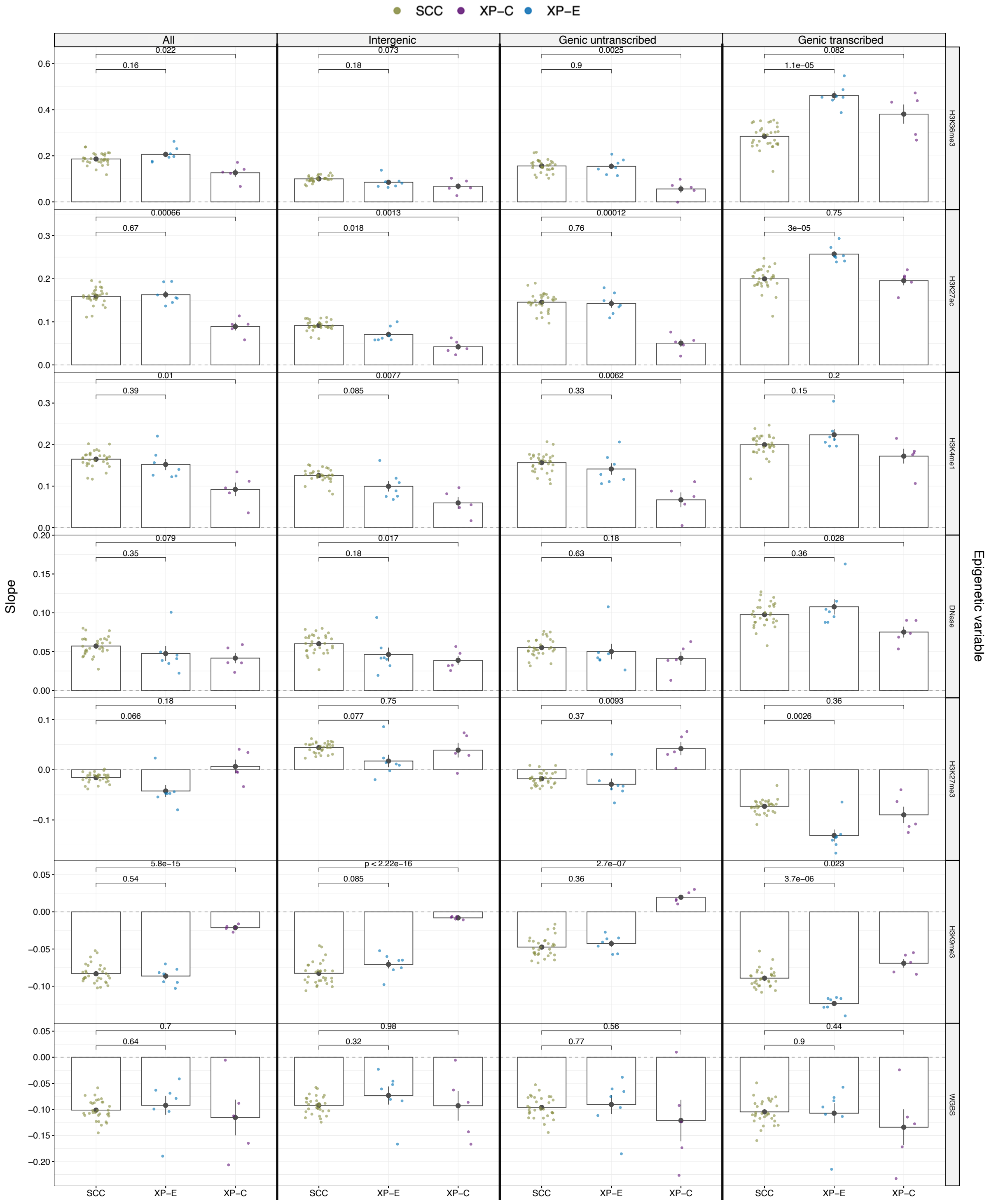

Supplementary Figure 9. The slope values from linear regressions across C>T mutations from pyrimidine dimers over binned epigenetic features for the whole genome (left panel), intergenic regions (left middle panel), untranscribed (right middle panel), and transcribed (right panel) strands of genes separately (only cSCC from sporadic, XP-E and XP-C groups were used in the analysis). *P*-values based on the Welch two-sample t-test, two-sided comparisons between sporadic cSCC and cSCC from XP-C or XP-E groups are indicated. Multiple testing adjustment was not performed. Data are presented as mean values +/- SEM. n = 31 for SCC, n = 5 for XP-C and n = 7 for XP-E (tumors)

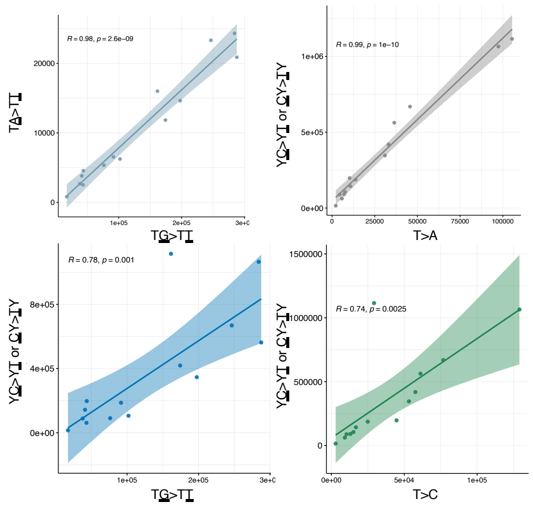

Supplementary Figure 10. Correlations between different types of substitutions in specific contexts in XP-V tumors. Pearson's r correlation coefficients and *P* values are indicated.

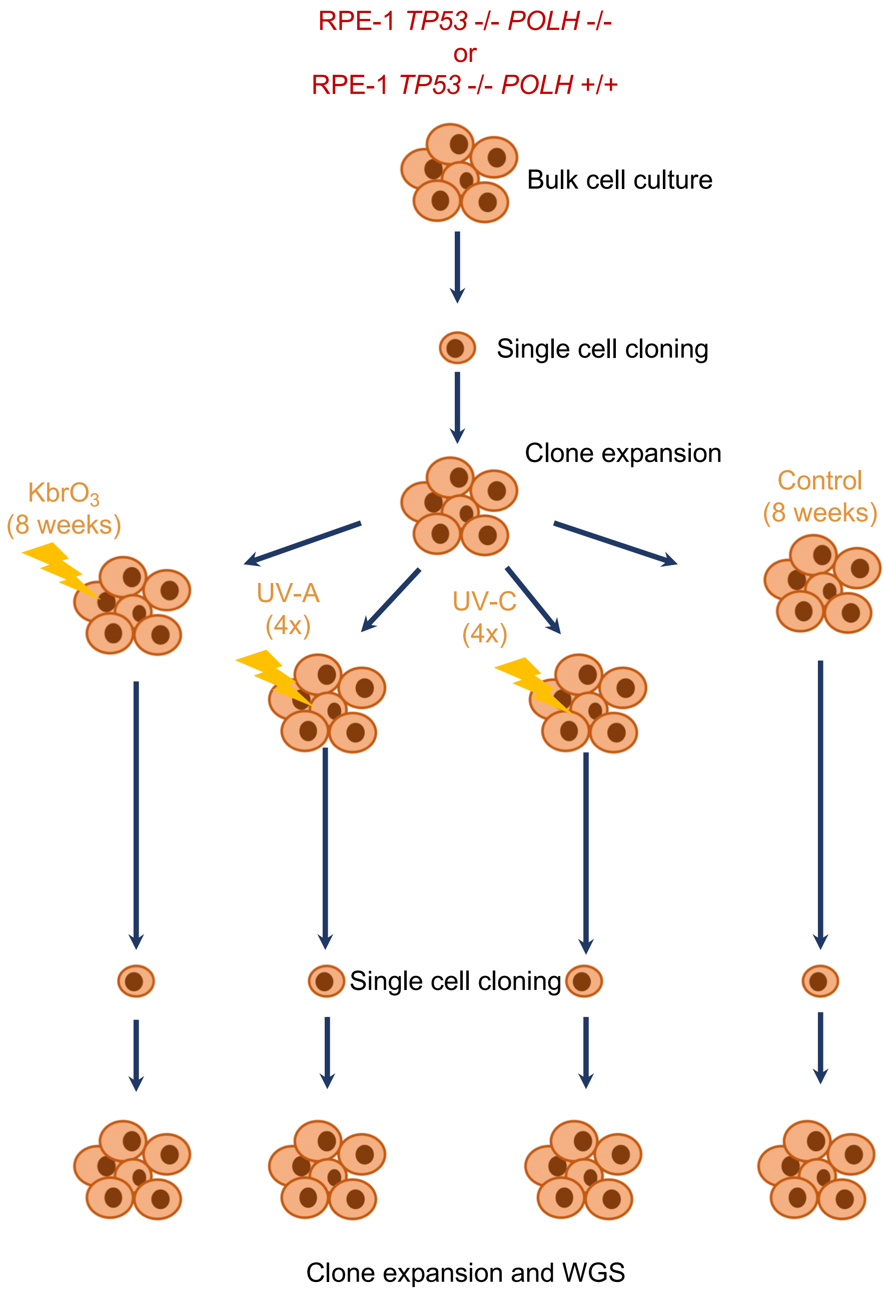

Supplementary Figure 11. Scheme of the mutation accumulation experiment with *POLH* KO and *POLH* wt cell lines.

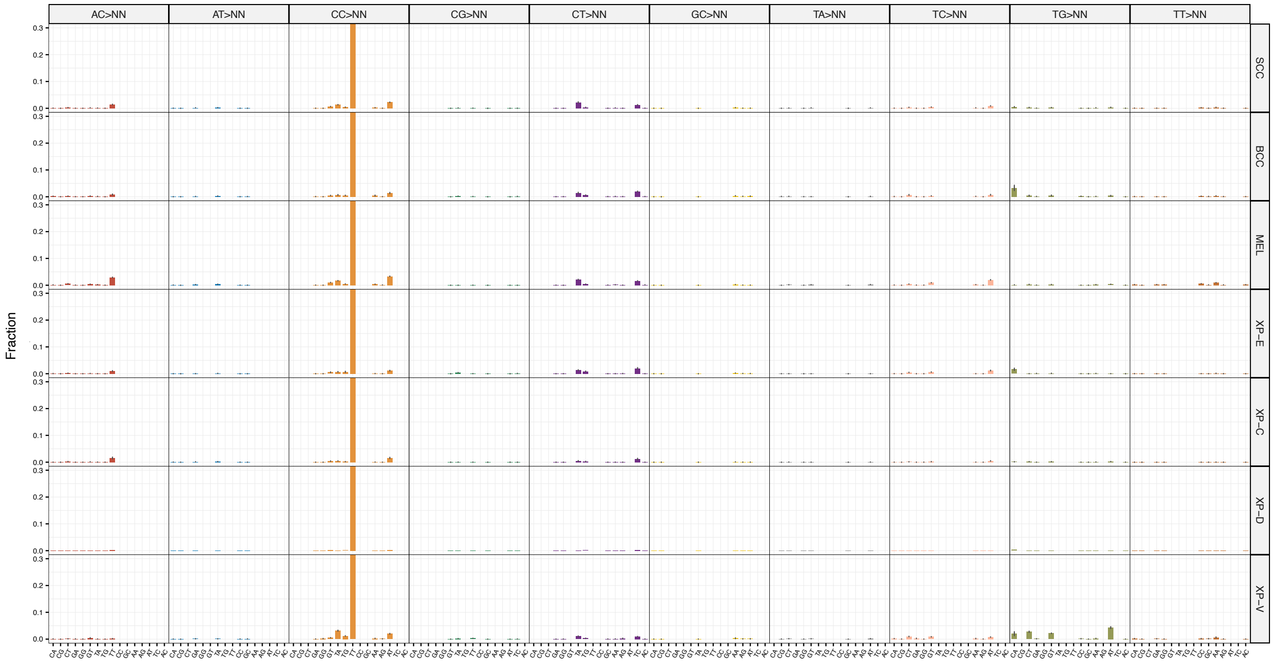

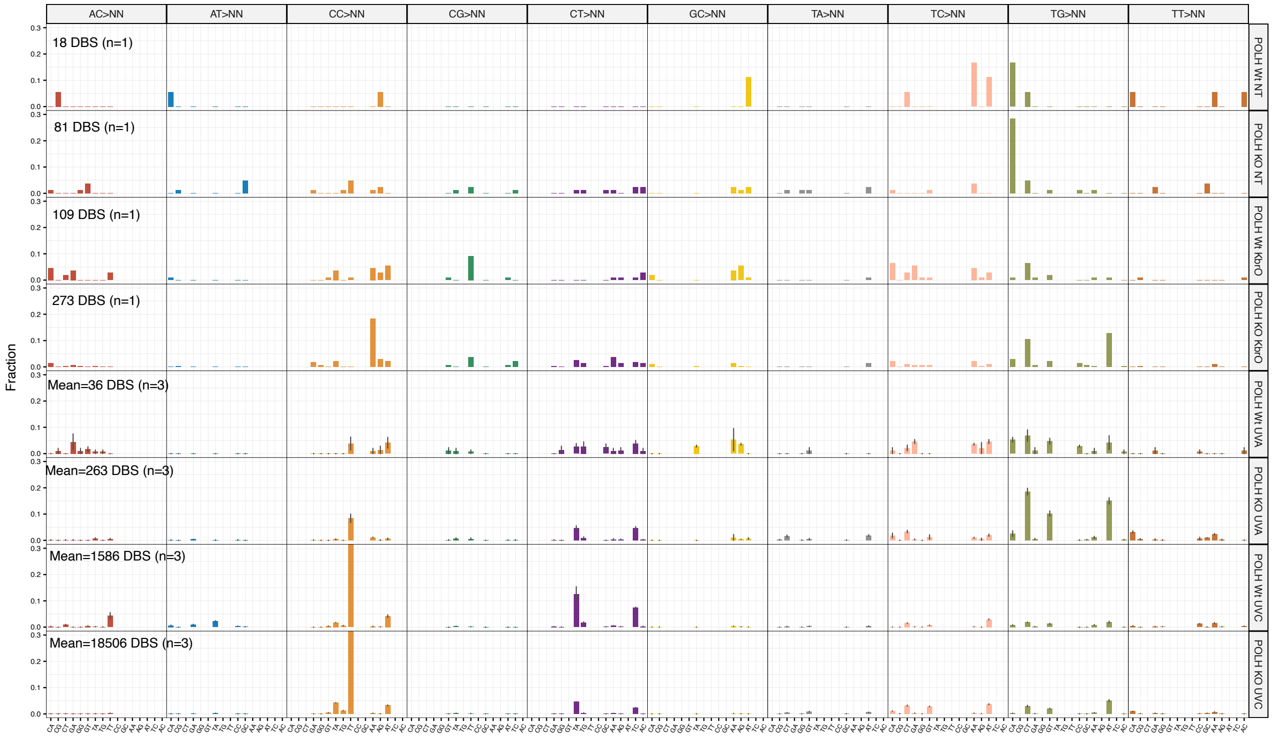

Supplementary Figure 12. Double base substitution (DBS) profiles of XP and sporadic skin tumors from fresh-frozen samples (upper panel) and RPE-1 mutation accumulation experiment (lower panel). Only fraction from 0 to 0.3 is shown. Data are presented as mean values +/- SEM. n – independent cell clones from RPE-1 cell line experiments. Sample size (upper panel, tumors): n = 31 for SCC, n = 8 for BCC, n = 113 for MEL, n = 10 for XP-E, n = 8 for XP-C, n = 3 for XP-A, n = 3 for XP-D and n = 14 for XP-V.

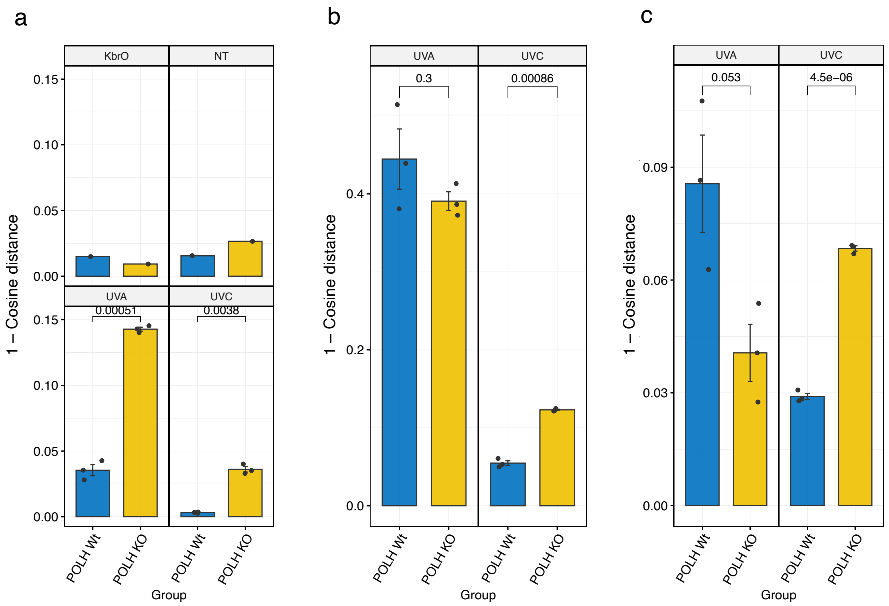

Supplementary Figure 13. Reconstruction of RPE-1 mutational profiles with COSMIC mutational signatures.

Cosine dissimilarity (1-Cosine distance) between original and reconstructed trinucleotide-context mutation profiles using:

a all the COSMIC mutational signatures and 96-chanell mutational profiles

b SBS7a/b/c/d COSMIC mutation signatures for all SBS. Data are presented as mean values +/- SEM.

c SBS7a/b/c/d COSMIC mutation signatures for C>T mutations with adjacent pyrimidine only. Significance was assessed with Welch two sample t-test, two-sided. Data are presented as mean values +/- SEM.

Sample size for all the panels (independent cell clones per cell line): NT, n = 1; treated with KbrO3 n = 1; treated with UV-A, n = 3; treated with UV-C, n =3.

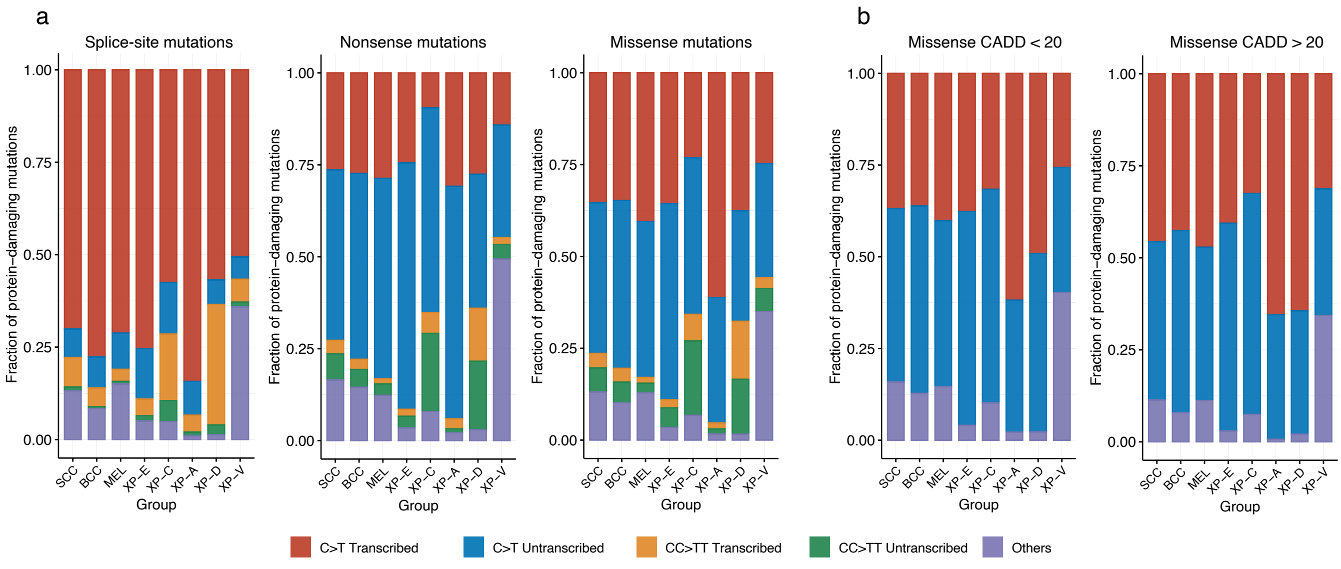

Supplementary Figure 14. Protein-damaging effect of mutation contexts for different classes of protein-damaging mutations.

a Mean fraction of protein-damaging mutations originating from the main mutation classes split by gene strand per group for missense, nonsense and splice-site mutations

b Mean fraction of protein-damaging missense mutations originating from C>T substitutions and others SBS split by gene strand per group for conservative (CADD > 20) and non-conservative (CADD < 20) groups.

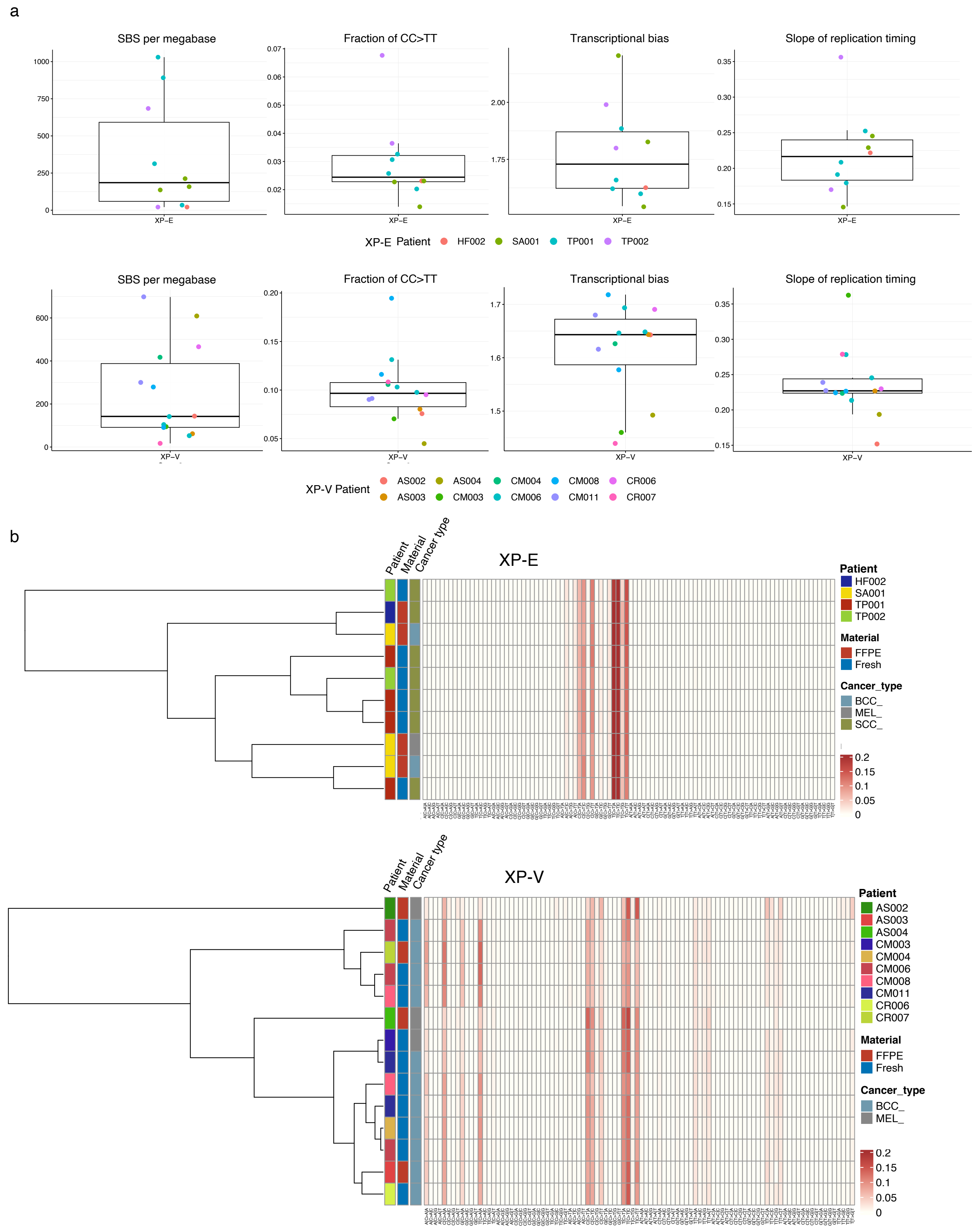

Supplementary Figure 15. Assessment of sampling from the same individuals for XP-E and XP-V groups.

a Visualization of individual samples from patients for XP-E (upper panel) and XP-V (lower panel) groups. Tumors taken from a single patient have similar colors. Boxes depict the interquartile range (25–75% percentile), lines - the median, whiskers - 1.5× the IQR below the first quartile and above the third quartile. n = 10 for XP-E and n = 14 for XP-V (tumors).

b Hierarchical clustering based on the Cosine similarity distance for XP-E (upper panel) and XP-V (lower panel) groups. Patient ID, sample material and skin cancer type are indicated by color codes.

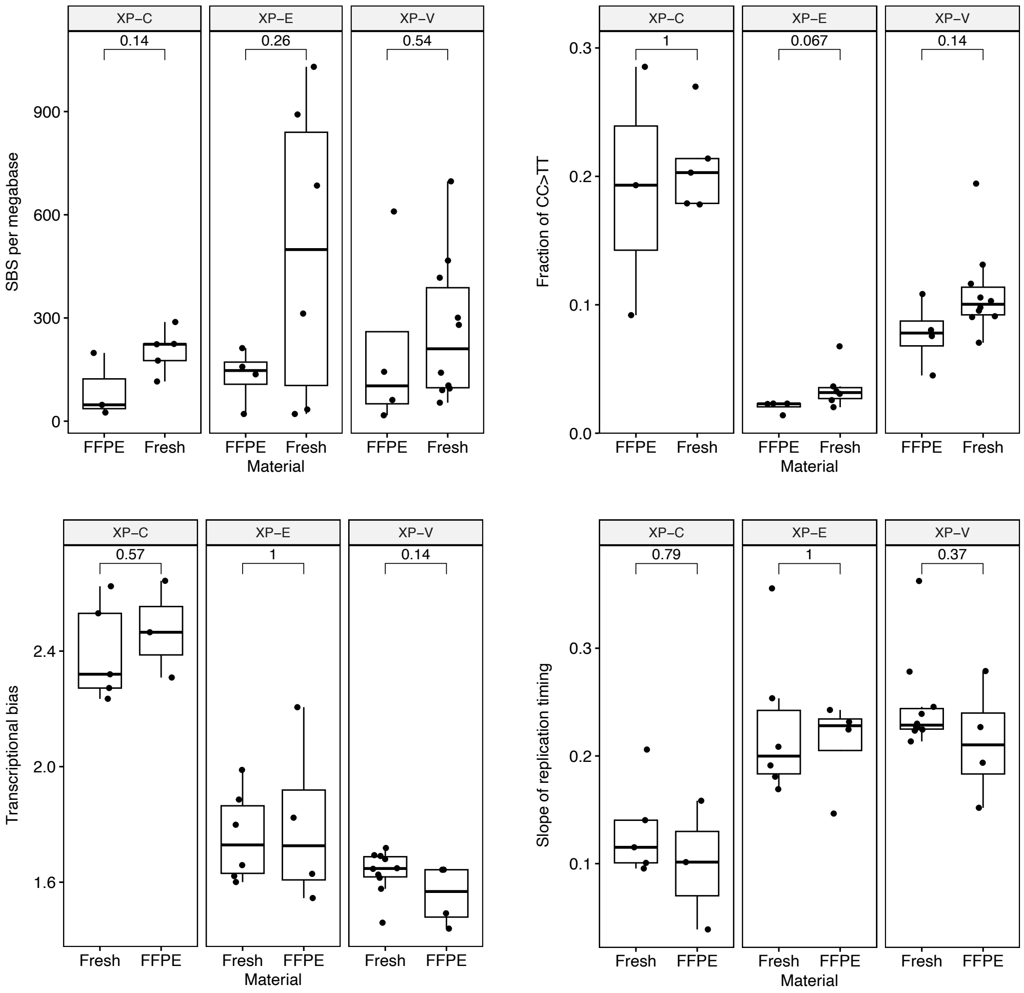

Supplementary Figure 16. Comparison between FFPE-derived and non FFPE-derived samples for the main analysis of the study (Performed only for groups with the sufficient sample size for statistical testing). Significance was assessed using Mann–Whitney U test, two-sided. Boxes depict the interquartile range (25–75% percentile), lines - the median, whiskers - 1.5× the IQR below the first quartile and above the third quartile. Sample size (tumors): n = 3 for XP-C FFPE, n = 5 for XP-C Fresh, n = 4 for XP-E FFPE, n = 6 for XP-E Fresh, n = 4 for XP-V FFPE and n = 10 for XP-V Fresh.

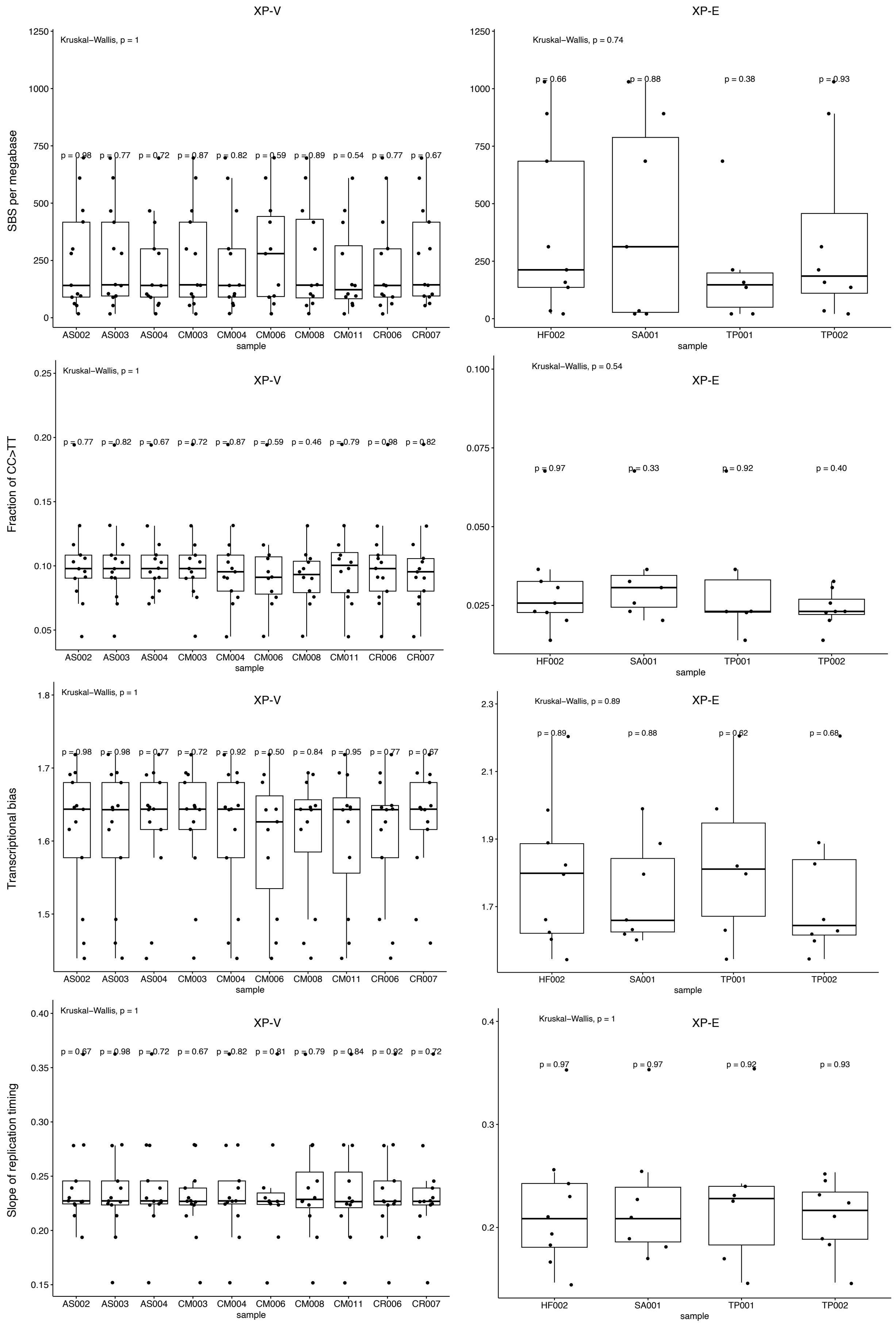

Supplementary Figure 17. Resampled datasets generated by removal of all the tumors from a single patient (excluded patient code is indicated on the x-axis; Kruskal–Wallis H test was used to produce global *P*-values). Boxes depict the interquartile range (25–75% percentile), lines - the median, whiskers - 1.5× the IQR below the first quartile and above the third quartile. Initial sample size (tumors:) n = 14 for XP-V and n = 10 for XP-E.
