# Supplementary material for: Genomic mutation landscape of skin cancers from DNA repair-deficient xeroderma pigmentosum patients
Source: Nat Commun. 2023 May 4;14:2561. doi: 10.1038/s41467-023-38311-0 (PMC10160032; doi:10.1038/s41467-023-38311-0)
Supplement: Supplementary file 3 — Reporting Summary [file 41467_2023_38311_MOESM3_ESM.pdf]

Corresponding author(s): Sergey Nikolaev
Last updated by author(s): Apr 17, 2023

Reporting Summary

Nature Portfolio wishes to improve the reproducibility of the work that we publish. This form provides structure for consistency and transparency in reporting. For further information on Nature Portfolio policies, see our Editorial Policies and the Editorial Policy Checklist.

Statistics

For all statistical analyses, confirm that the following items are present in the figure legend, table legend, main text, or Methods section.

n/a	Confirmed	
☐	☒	The exact sample size (*n*) for each experimental group/condition, given as a discrete number and unit of measurement
☐	☒	A statement on whether measurements were taken from distinct samples or whether the same sample was measured repeatedly
☐	☒	The statistical test(s) used AND whether they are one- or two-sided<br>*Only common tests should be described solely by name; describe more complex techniques in the Methods section.*
☐	☒	A description of all covariates tested
☐	☒	A description of any assumptions or corrections, such as tests of normality and adjustment for multiple comparisons
☐	☒	A full description of the statistical parameters including central tendency (e.g. means) or other basic estimates (e.g. regression coefficient) AND variation (e.g. standard deviation) or associated estimates of uncertainty (e.g. confidence intervals)
☐	☒	For null hypothesis testing, the test statistic (e.g. *F*, *t*, *r*) with confidence intervals, effect sizes, degrees of freedom and *P* value noted<br>*Give P values as exact values whenever suitable.*
☒	☐	For Bayesian analysis, information on the choice of priors and Markov chain Monte Carlo settings
☒	☐	For hierarchical and complex designs, identification of the appropriate level for tests and full reporting of outcomes
☐	☒	Estimates of effect sizes (e.g. Cohen's *d*, Pearson's *r*), indicating how they were calculated

Our web collection on statistics for biologists contains articles on many of the points above.

Software and code

Policy information about availability of computer code

Data collection	No software was used for data collection
Data analysis	QC of the reads: FASTQC (Andrews, 2015) (v0.11.7); Read mapping: BWA-MEM (v0.7.12) software (Li and Durbin, 2009), Bowtie 2 (v. 2.5.1) software (Langmead et al. 2012); BAM files preparation: samtools (Li et al., 2009) (v1.9); BAM QC: and multiqc (Ewels et al., 2016) (v1.5); variant calling: GATK mutect2 (Depristo et al., 2011) (v4.0.10.1); annotation of the variants: oncotator (Ramos et al., 2015) (v1.9.9.0), mutational signature analysis: MutationalPatterns software (Blokzijl et al., 2018) (v.1.11.0), SigProfilerMatrixGenerator v.1.0 software (Bergstrom et al., 2019), R statistical software v3.5.1; genomic coordinates: BEDOPS v2.4.37 (bedmap) software (Neph et al., 2012), BEDTools v2.30.0 (Quinlan, 2014); SCNA analysis: FACETS v0.5.14 (Shen and Seshan, 2016); pipeline assembler: snakemake v5.4.0 (Köster and Rahmann, 2012).

For manuscripts utilizing custom algorithms or software that are central to the research but not yet described in published literature, software must be made available to editors and reviewers. We strongly encourage code deposition in a community repository (e.g. GitHub). See the Nature Portfolio guidelines for submitting code & software for further information.

Data

Policy information about availability of data

All manuscripts must include a data availability statement. This statement should provide the following information, where applicable:

- Accession codes, unique identifiers, or web links for publicly available datasets
- A description of any restrictions on data availability
- For clinical datasets or third party data, please ensure that the statement adheres to our policy

Experimental WGS FASTQ files from tumors and normal tissue of patients generated in this study and corresponding filtered VCF files have been deposited in the European Genome-phenome Archive (EGA) under accession code EGAS00001006732 [https://ega-archive.org/studies/EGAS00001006732]. The raw FASTQ files and filtered VCF files are available under restricted access due to data privacy laws, and will be made available under approval by the data access committee [https://ega-archive.org/dacs/EGAC00001002945]. Access will be provided within approximately four weeks and be available for six months. Experimental sequencing data (WGS, FASTQ files) from the RPE-1 cell line experiments generated in this study have been deposited to NCBI under accession code PRJNA940340 [https://www.ncbi.nlm.nih.gov/bioproject/PRJNA940340] and corresponding VCF files are available on Mendeley Data server (DOI:10.17632/jkjkpvgxyd.1) [https://data.mendeley.com/datasets/jkjkpvgxyd]. These data are freely available.
The previously published melanoma samples from sporadic patients17 referenced in the study (consensus VCF files with SNVs and INDELs) are available in a public repository from the https://dcc.icgc.org/repositories website. Genomic datasets of XP-C and sporadic cutaneous SCC12 used in this study is available in the dbGaP database under accession code phs000830.v1.p1 [https://www.ncbi.nlm.nih.gov/gap/advanced_search/?TERM=phs000830.v1.p1], access is restricted and can be granted under approval by the data access committee. Previously published WGS FASTQ datasets of sporadic cutaneous SCC and BCC used in this study and provided by Hartwig Medical Foundation15 under data request DR-108 are available under restricted access after approval of the data sharing committee [https://www.hartwigmedicalfoundation.nl/en/data/data-acces-request/]. Previously published VCF files from the WGS study of metastatic cutaneous SCC16 are available under restricted access after approval of the data sharing committee (EGAS00001003370) [https://ega-archive.org/studies/EGAS00001003370]. Previously published XP-C angiosarcoma13 somatic variants are freely available on Mendeley Data server: 10.17632/7cxt72pckw.1 [https://data.mendeley.com/datasets/7cxt72pckw/1]. COSMIC database (v.3.2) of mutational signatures was used for the comparison of mutational profiles with previously identified mutational signatures (https://cancer.sanger.ac.uk/signatures/). Source data are provided with this paper.

Human research participants

Policy information about studies involving human research participants and Sex and Gender in Research.

Reporting on sex and gender	We do not report sex in this study.
Population characteristics	The study participants were patients with Xeroderma pigmentosum and skin cancers. This is a very rare hereditary disease and diagnosis of XP and skin cancer were the major indication for the inclusion to the study.
Recruitment	The participants were approached during routine clinical procedures. There was no specific selection of the patients except their diagnosis and consent. We do not anticipate a bias in recruitment of participants in this study
Ethics oversight	The samples were collected from patients with a confirmed XP diagnosis. Informed signed consents were obtained from patients and/or their parents per the Declaration of Helsinki and the French law. This study was approved by the French Agency of Biomedicine (Paris, France), the Ethics Committee from the CPP of the University Hospital of Bordeaux (Bordeaux, France), the Institutional Review Board of Gustave Roussy (CSET: 2018-2820; Gustave Roussy, Villejuif, France), the Research Ethics Committee of Guy's and St Thomas' Foundation Trust, London (reference 12/LO/0325), and the CONEP (Brazil), Number CAAE 48347515.3.0000.5467.

Note that full information on the approval of the study protocol must also be provided in the manuscript.

Field-specific reporting

Please select the one below that is the best fit for your research. If you are not sure, read the appropriate sections before making your selection.

☒ Life sciences ☐ Behavioural & social sciences ☐ Ecological, evolutionary & environmental sciences

For a reference copy of the document with all sections, see nature.com/documents/nr-reporting-summary-flat.pdf

Life sciences study design

All studies must disclose on these points even when the disclosure is negative.

Sample size	We did not predetermine sample size. All the available material was used in the study.
Data exclusions	We excluded sporadic skin cancer samples which demonstrated low enrichment of UV-induced mutations (less than 70%).
Replication	We did not attempt to replicate our results based on the sequencing of XP tumors because this is a rare disease and material is not available. Only independent biological replicates were used in this study. No technical replicates were used.

Randomization | The groups were allocated according to the diagnosis (corresponding XP group)

Blinding | The blinding was not applicable to our data. All the researchers acknowledged the grouping of the samples. The blinding was not relevant for this study because we worked with a collection and analysis of rare samples and their comparison with published cohorts.

Reporting for specific materials, systems and methods

We require information from authors about some types of materials, experimental systems and methods used in many studies. Here, indicate whether each material, system or method listed is relevant to your study. If you are not sure if a list item applies to your research, read the appropriate section before selecting a response.

Materials & experimental systems

n/a	Involved in the study	
☒	☐	Antibodies
☐	☒	Eukaryotic cell lines
☒	☐	Palaeontology and archaeology
☒	☐	Animals and other organisms
☒	☐	Clinical data
☒	☐	Dual use research of concern

Methods

n/a	Involved in the study	
☒	☐	ChIP-seq
☒	☐	Flow cytometry
☒	☐	MRI-based neuroimaging

Eukaryotic cell lines

Policy information about cell lines and Sex and Gender in Research

Cell line source(s) | RPE1 TP53-KO POLH-WT cell line was was obtained as a gift from Dr. Olivier Gavet lab (Institut Gustave Roussy, France). RPE1 TP53-KO POLH-KO cell line was generated based on the RPE1 TP53-KO POLH-WT cell line by Synthego company (CA, USA).

Authentication | None of the cell lines were authenticated

Mycoplasma contamination | The cells were checked by the Synthego company which performed POLH knockout using PCR methodology. All cell lines were mycoplasma negative. We did not check for contamination in our lab after receiving the cell lines.

Commonly misidentified lines (See ICLAC register) | No commonly misidentified cell lines were used in the study.
